# Research on the negative effect of product scarcity appeals on the purchase intention of green products and its mechanism

**DOI:** 10.3389/fpsyg.2024.1225011

**Published:** 2024-04-08

**Authors:** Shenghong Ye, Guangrui Liu, Yanfeng Lin, Zhiheng Lin, Yijing Shi, Zan Huang

**Affiliations:** School of Management, Jinan University, Guangzhou, China

**Keywords:** product scarcity, perception of deceptive, perceived green washing, theory of persuasion knowledge, motivation of impression management

## Abstract

Studies have shown that product scarcity appeals affect consumers’ perceived scarcity, willingness to pay, and other responses, and that scarcity appeal has the potential to cause consumers to pay higher attention to the product. However, there is a lack of research on the psychological responses of consumers to scarcity appeal from the perspective of perceived green washing. In this paper, three experiments are conducted to demonstrate the impact of product scarcity appeals on consumers’ purchase intentions. The research shows that when green products use product scarcity appeals as a strategy, consumers’ purchase intentions are affected, but consumers’ information processing about the product is the most important determinant. Perceived green washing mediates the negative effect of product scarcity appeals on green product purchase intentions. And impression management motives moderate the negative effect of product scarcity appeals on green product purchase intentions. The findings of the study not only help companies to effectively adopt the right advertising strategies to improve their marketing effectiveness, but also help them to explore the market for green products.

## 1 Introduction

In today’s society, people gradually pay attention to the safety and health of the quality of life, and pay more attention to the relationship between consumer quality and sustainable development in the process of consumption. In fact, green consumption has become a core and enduring movement globally, one that is likely to strengthen as younger generations grow up and buy more green products from businesses at a higher rate than previous generations (Lu et al., 2013). Many companies have taken note of these requirements and responded positively to them by producing and offering green products that balance corporate, consumer, social and ecological interests and green marketing to meet the green needs of consumers. For example, in 2016, 190 Fortune 500 companies reported that they benefited from $3.7 billion in green products (WWF et al., 2017). Therefore, there are more and more green products on the market to meet the needs of consumers. Green products are those products that do not harm the environment and do not contain potentially harmful components, specifically, products that do not pollute the environment or destroy natural resources, can be recycled or can save energy (Lin and Chang, 2012). Compared with ordinary products, green products have considerable pro-social properties (Wang et al., 2019). Correspondingly, green consumption behavior refers to consumers’ daily consumption, which focuses on purchasing products that can satisfy their intrinsic needs while reducing the negative impact on the environment (Mao et al., 2017).

However, most consumers still have a weak awareness of ecological protection, and it is difficult to judge whether the product is green washing or not. Consumers also tend to have attitude-behavior differences in green consumption behavior, i.e., inconsistencies between individual attitudes toward green products and actual purchase behavior. In light of this, current research in the area of green consumption continues to explore how to motivate consumers to adopt green products. For example, there has been a lot of interest in how businesses can use effective strategies to communicate their messages to motivate consumers to respond positively to green products. Previous research has focused on advertising appeals, such as rational versus emotional appeals, self-interest versus altruistic appeals, guilt appeals, and concrete versus abstract appeals (Kareklas et al., 2012; Lin and Chang, 2012; Matthes and Wonneberger, 2014; Yang et al., 2015; Mao et al., 2017). It is worth mentioning that most studies mainly focus on the promotion effect of advertising appeals on green products, and few studies pay attention to their negative effects. Meanwhile, product scarcity appeals, as a common type of advertising appeals in marketing practice, have received more attention in the field of advertising research, and related studies have found that product scarcity appeals are a frequent promotional strategy used by businesses to attract consumers’ attention and interest (Liu and Li, 2017; Chae et al., 2020), however, by combing through the literature on green marketing, it is found that its role in the advertising and marketing of green products is still less explored.

In addition, there are now studies on the mechanisms of consumer responses to product scarcity appeals in terms of perceived scarcity and psychological resistance. However, there are still relatively few relevant studies, and the lack of integrated research on consumers’ psychological reactions to scarcity appeals from the perspective of perceived green washing is undoubtedly limiting the effective insight into the nature and comprehensiveness of the consumer scarcity effect. Product scarcity appeals can have an important impact on consumers’ consumption motivation and behavior. Previous studies have found that when consumers are faced with a product with scarcity appeals, on the one hand, consumers’ perceived competitiveness is aroused, thus prompting them to make promotional purchases; on the other hand, as consumers’ persuasion knowledge increases, consumers’ perception of deceptive is also aroused, thus prompting them to make defensive purchases. Previous studies have found that consumers’ “sense of freedom” is awakened by the scarcity of information, as they feel restricted in their freedom of choice, which leads to stronger psychological resistance and reduced willingness to buy (Li D.J. et al., 2015; Li D.Y. et al., 2015).

In this study, the effects of product scarcity appeals will be discussed. In detail, this study will focus on product scarcity appeals, perceived green washing, and motivation of impression management for green products, specifically exploring the following issues: (1) What is the role of product scarcity appeals in green product advertising and marketing? (2) If there is an effect, what is the potential mechanism?

In this paper, the product scarcity appeals were manipulated by designing two different subgroups, the “scarcity group” and the “control group.” Then, a marketing scenario was described in which the subjects were asked to evaluate their willingness to buy two products, and then they were asked to fill in the questionnaire items related to perceived green washing and motivation of impression management to reflect their psychological characteristics and willingness to buy green products with product scarcity appeals.

This paper has several managerial insights and theoretical contributions to the business and existing literature, respectively. First, this paper utilizes an experimental approach to investigate the mechanisms that influence consumers’ willingness to purchase green products that use product scarcity appeals as a strategy. The study not only helps firms to effectively adopt the right advertising strategies to improve marketing effectiveness, but also helps them to explore the green market. The purchase of green products is one of the main ways to promote the green development of society, protect the environment and transform the traditional lifestyle of consumers into “green life.” Therefore, the research of this thesis adds a certain theoretical basis for promoting the construction of ecological civilization and for consumers’ participation in green consumption.

In addition, academic research on advertising appeals in green marketing currently focuses on several types of advertising appeals, such as self-interest and altruism appeals, rational-emotional appeals, guilt appeals, and concrete and abstract appeals, while product scarcity appeals, which are common in marketing practice, have been less explored. This study examines the effect of product scarcity appeals on the purchase intention of green products in a green product purchase context; meanwhile, while previous studies have mainly explored the positive effects of advertising appeals, this study examines the negative effects of a common type of advertising appeals in practice, product scarcity appeals. This study enriches the existing research on product scarcity appeals.

### 1.1 Literature review and hypotheses development

#### 1.1.1 The influence of product scarcity appeals on the purchase intention of green products

Product Scarcity Appeals (PSA) refers to the use of advertising or marketing to increase the attractiveness of a product, giving the public the idea that the product is in short supply in order to arouse consumers’ attention and purchase (Eisend, 2008; Aguirre-Rodriguez, 2013). Merchants usually express the scarcity appeal of a product by highlighting the shortage of the quantity of the product. It is found that when the demand for a good increases and the quantity of the good is short, the public who want to buy the good will increase their value judgment and purchase urgency (Xu et al., 2021). For example, merchants often use hunger marketing, special offers, and limited supply to create an atmosphere of oversupply of goods, so that the public cannot immediately own the goods while making the public aware of their popularity. Numerous studies have found that product scarcity appeals increase the value of the product while promoting the sales of scarcity type products (Bozzolo and Brock, 1992), thus leads to greater product expectations, more purchases, shorter search times and higher purchase satisfaction (Xu et al., 2021), and of course, more positive willingness to pay and purchase (Eisend, 2008; Huang et al., 2020). However, it has also been shown that publicizing the oversupply of a commodity does not cause the public to pay attention to the extent of the commodity and its value assessment, and that the exact effect depends on the cause of the scarcity (Lazaroiu et al., 2019) and the consumer’s information processing of scarcity appeals (Eisend, 2008; Aguirre-Rodriguez, 2013). The reasons for the increase in demand for goods include environmental and human factors; the increase in demand due to environmental factors is difficult to promote the purchasing behavior of the public, and the increase in demand due to human factors tends to make the public willing to purchase and purchase behavior (Verhallen and Robben, 1994). At the same time, consumers with purchasing power are difficult to increase their willingness to purchase and consumers with weaker purchasing power are more likely to generate purchase ideas (Xu et al., 2021). Even the increase in demand due to artificial factors has different purchasing effects on the general public. For example, for the case where the supply side does not change the supply of the good and for the case where the product cannot meet the market demand, the two have different scarcity effects (Verhallen and Robben, 1994). In the case of intentional limited supply, consumers may experience perceived green washing and psychological resistance, and the consumer’s desire for exclusivity or uniqueness is reduced (Román and Ruiz, 2005). Thus, product purchase behavior for two different reasons of scarcity and types of scarcity can have different effects on consumers’ value judgments and purchase intentions.

Rational and emotional needs, altruistic needs, and weighing environmental benefits are mainly used in the advertising of green products. Yang et al. (2015) suggest that abstract advertising can be used to emphasize altruistic benefits of green products, thus increasing consumers’ desire to purchase. However, studies such as Darke and Ritchie (2007) and Riquelme and Román (2014) found that as consumers become more experienced and their persuasion knowledge increases, they are increasingly likely to process incoming advertising messages defensively and thus are more likely to develop perception of deceptive. It has also been shown that consumers are also increasingly inclined to speculate on the intentions behind the operation of scarcity advertisements, and if perceived deceptive will thereby reduce their willingness to purchase the product (Chaouachi and Rached, 2012; Mukherjee et al., 2012). Consumers are more likely to be influenced by information dissemination factors in the process of making purchase decisions on green products (Lin and Chang, 2012). Hence, consumers facing green product advertisements that express product scarcity appeals are more likely to be deceived and resistant, thus reducing their desire to buy green products (Majerova et al., 2020).

Persuasion knowledge theory refers to the personal knowledge that consumers acquire over time to reasonably respond to various persuasion strategies of marketers in order to ultimately achieve their goals (Friestad and Wright, 1994). Once consumers have more persuasion knowledge, they can more easily identify, analyze, interpret, evaluate, and remember the intent of various persuasions, and then choose and implement responses that they consider appropriate and effective, thus reducing the likelihood of being deceived. After the emergence of the theory of persuasion knowledge, relevant studies explaining consumer behavior gradually shifted from a passive perspective to a focus on individual initiative. In fact, Mobbs et al. (2020) found that consumers confronted with exposed information engage in both precision and defensive processing. Defensive processing makes consumers trust the information less. Some studies have confirmed that consumers process information defensively mainly due to two aspects. One of them is to avoid the aggression that comes from advertising messages (Darke and Ritchie, 2007); on the other hand, it stems from consumers’ aversion to risk, and they will avoid losses caused by not being able to accurately identify deceptive messages. Therefore, it has been confirmed that when persuasion knowledge is enhanced by defensive focus, consumers tend to be wary of being deceived by manipulative advertising deception. Product scarcity appeals, on the other hand, are often a distinctive feature of virtual products in virtual product consumption contexts. Due to the vague product concept and lack of physical presence, consumers perceive greater risk and are thus more prone to perception of deceptive in certain contexts (Li D.J. et al., 2015; Li D.Y. et al., 2015; Liu et al., 2017). Research in areas related to green products, Lee et al. (2015) in the process of exploring the dissemination of product scarcity appeals, also pointed out that if consumers subjectively perceive signs that corporate activities will intentionally lead them to consume, they will have negative evaluations of corporate products, and consumers are largely influenced by emotions in the process of purchasing green products, and the expression of scarcity appeals for green products are more likely to play a negative role in consumers’ willingness to purchase. Scholars such as Aguirre-Rodriguez (2013) argue that once consumers have a higher level of persuasion knowledge, they are less likely to favor purchasing goods that have advertising scarcity appeals. Specifically in the case of green product purchases, the expected guilt generated by consumers not purchasing green products due to self-responsibility is diminished, thus reducing purchase intentions.

In summary, the following hypotheses are proposed in this paper:

*H*1: Product scarcity appeals will have a negative effect on the willingness to purchase green products.

### 1.2 The mediating role of perceived green washing

The term “green washing” is a hybrid of the words “green” and “whitewash,” and is a proxy for false corporate environmental claims as well as whitewashing practices (Bowen and Aragon-Correa, 2014). In the 1990s, the concept of “green consumption” became prevalent in developed countries such as the United States, and many companies began green washing in response to this market trend. Over the past few years, green washing has become a common occurrence in companies due to a combination of the market’s move toward a green economy, the prevalence of green concepts, and inadequate regulation (Lyon and Montgomery, 2015). Some of the representative greenwashing activities include targeting the company’s achievements in environmental protection to hide the pollution generated in the production process, or falsely reinforcing the positive impact of the product’s green features on the environment (Li D.J. et al., 2015; Li D.Y. et al., 2015). In addition, one-sided glittering generality and “verbal environmental protection” are also common tactics that consumers can perceive as corporate greenwashing behavior (Yang et al., 2020; Huang and Lei, 2021). With the emergence of corporate green washing, it makes consumers think more when they are confronted with green perceived marketing messages and will discern the authenticity of green messages. When most consumers believe that green products are a marketing strategy used by companies, their perceived green washing increases and they do not believe the information provided by companies that the products are “green.” Consumers’ perceived green washing, i.e., their judgment of the authenticity of green information, will affect their consumer attitudes and their attention to green products. In essence, perceived green washing is also a kind of perception of deceptive, and most existing studies have explored the negative effects of perceived green washing on consumers’ green purchasing behavior from the perspective of green purchasing. For example, Wu (2014) showed that perceived green washing affects consumers’ perceived green quality and green satisfaction, and ultimately affects consumers’ green word of mouth, which is an important factor influencing consumers’ purchase intentions. Nyilasy et al. (2014) found that when there is a role between the interaction of green advertising and the environmental performance of a company, the environmental performance of a merchant is lower and consumers will initiate perceived green washing and believe that the green information provided by that merchant is false, which also has a significant negative impact on brand attitudes. Chen and Chang (2013) and other scholars found that consumers’ perceived green washing not only increases confusion and green perception risk in green consumption, but also has a significant impact on trust in green products. Therefore, the level of consumers’ perceived green washing affects their green purchase intentions; the lower the perceived green washing, the higher the consumers’ purchase intentions; the higher the perceived green washing, the lower the consumers’ purchase intentions.

Therefore, if green products are promoted using product scarcity appeals, consumers are likely to perceive such promotion as a strategy for companies to promote themselves as green and environmentally friendly to gain social reputation, rather than really wanting to produce green products, because these products are “scarce” or even high-priced, and consumers may think that companies do not want to produce them in large quantities for the sake of environmental improvement, and thus consumers may have perceived green washing, which affects their willingness to buy the products.

Based on the above analysis, the following hypothesis is proposed in this paper:

*H*2: Perceived green washing mediates the negative effect of product scarcity appeals on willingness to purchase green products.

### 1.3 The moderating effect of motivation of impression management

Grant and Mayer (2009) suggest that motivation of impression management plays a positive role in citizens engaging in pro-social behavior, and Finkelstein and Penner (2004) explain motivation of impression management as the expectation to maintain a positive external impression and to be present frequently in environmentally relevant scenarios. When consumers receive praise from others for their first experience with a green product, they are more likely to repeat the purchase repeatedly, thus receiving more praise to improve their image in the eyes of others. This is because most consumers perceive their image as being “dignified” when they engage in such activities and, conversely, “disgraced” when they do not receive positive comments (Goffman, 2023). Whether or not one is dignified is an important point that people give importance to under the influence of Confucianism (Dong and Zhang, 2024). Therefore, consumers are more inclined to behave in a pro-social manner for the purpose of maintaining “dignified” and thus receive external praise.

The purchase of green products can not only be praised by others, but can also help consumers to improve their moral quality by leaving a good image in their mind through such a purchase. Researchers such as Gierl and Huettl (2010) argue that consumers will increase their dependence on scarce products if they are satisfied with the conspicuousness of such products. This is because interpersonal comparisons in everyday life and the desire to be affirmed by others increase the need for consumers to influence their status in society by using and purchasing scarce products from which they receive compensation for their ego. Wu (2014) suggests that it is known from altruistic studies of green products that consumers will increase their demand for green products prompted by motivation of impression management. Therefore, if the motivation of impression management is strong, the chances of repeated purchase of green products by consumers will subsequently increase.

Motivation of impression management is inseparable from green consumption behavior. Green consumption behaviors (e.g., green product purchases) are pro-social behaviors (Wang et al., 2019), and Grant and Mayer (2009) point out that motivation of impression management drives people to make pro-social and altruistic moves. In addition, Griskevicius et al. (2010) state that consumers purchase green products because this behavior carries altruistic characteristics, which can show or maintain the status of the individual, among others. Because altruism reflects the motivation to bear the cost of expenses because of others, in non-private situations or when the price of green products is higher, consumers will be willing to purchase green products to improve their status. Taken together, people will conduct activities in accordance with socially esteemed codes of conduct and concepts because they are in the purpose of impression management, even sometimes at the expense of personal interests, in order to gain prestige and a good impression in the eyes of others (Finkelstein and Penner, 2004). Whereas purchasing green products is an easy path for consumers to get social praise and improve their image, consumers’ willingness to purchase green products changes because of the intensity of motivation of impression management (Wu, 2014), which also fits with the purpose of impression management.

In previous studies, it was concluded that most consumers are prone to impulsive consumption (Lee et al., 2015) because they are induced by a consumption philosophy that “scarcity means value” and that some consumers will consume excessively and competitively because of “scarcity” addiction. Barton et al. (2022) had found that consumers believe that buying so-called scarce products is a sign of self-efficacy and a symbol of a “savvy” shopper. Thus, in general, the scarcity appeal of green products can facilitate product purchase by consumers with high motivation of impression management in two ways. First, the purchase of green products is a pro-social act, and consumption of green products may lead consumers to believe that they will make a good impression on others or enhance their moral image, so consumers with high motivation of impression management may prefer green products promoted with scarcity appeals to those with low motivation of impression management, and perceived green washing is lower. At the same time, “scarce” green products are often in short supply and can, to some extent, reflect a consumer’s uniqueness or even status, so consumers with high motivation of impression management may prefer green products promoted with scarcity appeals and have lower perceived green washing than those with low motivation of impression management.

Accordingly, this study proposes a second hypothesis:

*H*3: Motivation of impression management moderates the mediation of perceived green washing in product scarcity appeals on green product purchase intentions.

**Figure 1:**
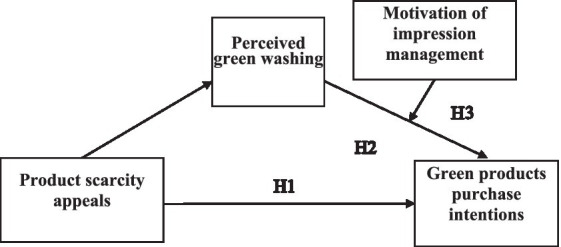
Conceptual model.

Figure 1 shows the complete conceptual model.

## 2 Pretest of the three experiments

The pretest was divided into two parts, which focused on the initial testing of the experimental stimulus materials, methods, and design that would be used in the three formal experiments that followed. In detail, the first part was to select valid stimuli for the later experiments. The second part was to ensure the validity of the methods and scales used in the formal experiments.

### 2.1 Experimental design and subjects

The whole experiment was divided into two parts, firstly, in the first part, before the formal experiment started, this study referred to the experimental process of Liu et al. (2017) and Wang et al. (2018) to determine the green products needed for the formal experiment. Detailed steps: firstly, through literature review and interviews, based on consumers’ daily life habits, products with scarcity appeals as advertising used in this study were identified as experimental materials. That is, through group talks, we selected five green products that consumers are more closely involved in their daily lives: recyclable paper bottles, recycled printing paper, biodegradable plastic, green paint, and environmentally friendly fabrics. In the second step, we recruited 30 college students from a university in southern China by means of a simple questionnaire to measure their definitions of the attributes of these products, the likelihood of daily purchases, and their judgments of whether these products were green. We referred to the scales of Liu et al. (2017) and Wang et al. (2018), translated the scales into Chinese, and invited scholars in related fields to suggest modifications. The survey results showed that the ratings of these products were 100, 80, 90, 85, and 95%. This led to the determination that these five products were identified as green products in everyday life. Finally, this thesis selected the highest rated eco-friendly fabrics, recyclable paper bottles and biodegradable plastics as stimuli to be measured in the subsequent experiments.

The second part was a pretest of the scarcity appeal, perceived green washing, motivation of impression management and purchase intention scales that will be covered in the main experiment. The material of the pretest was selected as the stimulus for the first part, which was “eco-friendly fabric.” 46 college students were recruited from a university in southern China and randomly divided into a scarcity group and a control group to read two materials about environmental fabrics. The experimental results of 40 valid subjects were obtained by this experiment after eliminating invalid samples (*M*_age_ = 24.38, *SD*_age_ = 3.53; 55% female). The contents of the materials were:

Control group: “fast fashion” provides the current popular styles and elements, it is characterized by low price, more models, more quantity, the current fiber clothing, chemical fibers have become an important part of the textile fiber. Common synthetic fibers are nylon, polyester, etc. They all obtain raw materials from petrochemicals to synthesize polymer, and then draw into fibers. Although there is some harm to the environment, but because the raw materials are cheap and easy to obtain, can stimulate the interest of consumers, the annual production of clothes are very considerable, can meet the market demand to the maximum extent.

Scarcity group: With the ultimate alarm bell ringing about environmental pollution and energy crisis, more and more eco-friendly clothes are appearing on the market. In order to redeem the negative image in consumers’ minds and to make current and future fashion more sustainable, a brand has set up an eco-friendly collection exclusively. It provides fashion for environmentally conscious customers, promotes concern for climate change, reduces waste, and reuses and recycles. The collection is designed to reduce the negative impact of clothing on the environment throughout its life cycle and to increase the recycling rate of textile fibers so that they can be fully reused. However, as the collection of old fabrics is limited in quantity and color, most of the processed eco-friendly clothes are limited edition.

### 2.2 Experimental procedures

After the subjects read the pairs of materials individually, they were asked to rate the product scarcity within the materials. If the product scarcity appeal rating for the scarcity group was lower than the product scarcity appeal rating for the control group, then this sample was of the control group type, and if it was higher then it was of the scarcity group type. To determine the validity of the experimental material, we used the questionnaire questions used in White and Peloza (2009) and Liu et al. (2017) studies and measured them on a 5-point Likert scale (1 = not at all scarce, 5 = very scarce). The questions on the motivation of impression management scale were “I want to present myself in a positive way to others” and “I want to make a positive impression on others” and so on (1 = strongly disagree, 5 = strongly agree; α = 0.799). Subjects were also asked to answer questions on the perceived green washing scale, such as “I think the product’s environmental promise is trustworthy” and “I think the product’s environmental appeals are trustworthy” (1 = strongly disagree, 5 = strongly agree; α = 0.864). In addition to the questions on the green product purchase intention scale, such as “I would recommend my relatives and friends to buy this product” and “I would like to collect and learn more information about this product” (1 = strongly disagree, 5 = strongly agree; α = 0.856). Statistical analysis of the reliability and validity of the scores given by the subjects to the scale items was carried out by SPSS 26, and the results were shown in Table 1, which showed that the reliability and validity of all the scale items were above the standard.

**Table 1:** Results of reliability and validity analysis.

Variables Liu et al. (2017) and White and Peloza (2009)	Items	Cronbach’s α	KMO	CR	AVE
Motivation of impression management	I want to present myself in a positive way to others	0.793	0.716	0.921	0.716
	I do not care what people think of me	0.784	0.743	0.936	0.725
	I want to make a positive impression on others	0.854	0.836	0.911	0.852
	I want to make myself look good to others	0.762	0.766	0.908	0.734
Perceived green washing	I think the product’s environmental promise is trustworthy	0.833	0.799	0.921	0.843
	I think the environmental performance of this product is reliable	0.818	0.822	0.918	0.801
	I think the product’s environmental appeals are trustworthy	0.764	0.742	0.943	0.746
	The product’s concern for the environment is in line with my expectations	0.739	0.803	0.883	0.728
	The product is committed to protecting the environment	0.798	0.747	0.908	0.832
Green product purchase intention	I would like to collect and learn more information about this product	0.821	0.838	0.919	0.811
	I would recommend my relatives and friends to buy this product	0.768	0.795	0.923	0.834
	I would love to introduce and recommend this product to my family	0.789	0.751	0.909	0.752
	I would buy this product if I needed to buy it	0.852	0.822	0.912	0.881

### 2.3 Results

#### 2.3.1 *T-*test

By performing an independent samples *t-*test on the two groups of data in the scarcity and control groups, the results show that there is no significant difference between the two groups in terms of perceived green washing (*t* = −1.018, *p* > 0.05); no significant difference in motivation of impression management (*t* = −0.343, *p* > 0.05); and a significant difference in purchase intention (*t* = 3.606, *p* < 0.05).

## 3 Experiment 1

The purpose of experiment 1 was to verify whether the negative effect of product scarcity appeals on green products purchase intentions holds true.

### 3.1 Experimental design and subjects

A (scarcity group vs. control group) single factor experimental design was used for experiment 1. The same experimental materials as well as methods were used as in the pretest, and 110 undergraduate students from a university in southern China were used as subjects in this experiment. The data of subjects who did not meet the requirement of filling in the data were eliminated according to the principle of completing normality, and a total of 100 valid sample numbers were finally obtained (*M*_age_ = 24.21, *SD*_age_ = 3.32; 54% female).

### 3.2 Experimental procedures

The experimental stimulus used in this thesis was an advertising message from the Internet and other media looking for relevant green products with product scarcity as appeals, thus forming the experimental material. The experiment began by randomly assigning 110 students from a university in the south to the two groups mentioned above, which were divided into a scarcity group and a control group. Then the purchase intention of the students in the two groups was measured separately by the purchase intention scale. Each subject participating in this experiment received a small gift after completing the experimental task independently. A marketing scenario was used to manipulate the subjects’ perceptions of product scarcity of green products by having them read a self-compiled material. The experimental starter materials for both the product control and scarcity groups were used from the pretest.

After reading the material, in order to determine the validity of the experimental material, subjects completed the questionnaire questions used in our study by White and Peloza (2009) and Liu et al. (2017) which were measured using a 5-point Likert scale. After reading the material, subjects continued to rate their perceived emotions on a five-point scale (1 = not at all scarce, 5 = very scarce). The subjects were also asked to answer the questions about their purchase intentions about green products in the questionnaire, and to choose the corresponding score according to their level of agreement after reading the materials and their level of agreement with each statement (1 = strongly disagree, 5 = strongly agree).

### 3.3 Results

Experiment 1 first divided the respondents into a scarcity group and a control group, and after excluding the invalid sample, the results of the data collected showed that there were 55 respondents in the scarcity group and 45 respondents in the control group. The hypotheses were tested by one-way ANOVA. In order to exclude the influence of control variables such as gender, age and education on the experimental results, we added gender, age and education together with product scarcity appeals as independent variables to the model. The significance of the corrected model *p* = 0.000 < 0.05 showed that the current model had independent variables that had a significant effect on the dependent variable. The model fit was 0.353, which met the general linear model general criteria. The results of the between-subjects effect test showed that product scarcity appeals had a significant effect on purchase intention in the model (*F* = 53.52, *p* = 0.000 < 0.05). From the mean comparison results, it was concluded that the mean value of willingness to purchase green products was lower in the scarcity group (*M*_scarcity_ = 2.261, *SD*_scarcity_ = 0.737; *M*_control_ = 3.336, *SD*_control_ = 0.727). Thus the negative effect of product scarcity appeals on purchase intention holds.

### 3.4 Discussion

Experiment 1 initially confirmed the relationship between product scarcity appeals and consumers’ purchase intentions by initiating subjects’ perceptions of product scarcity and testing that purchase intentions in this scenario were consistent with hypothesis 1 of this paper, which states that subjects have lower purchase intentions for green products with product scarcity appeals. But the specific reason for the relationship between the two is unclear. In this regard, Experiment 2 was designed in this paper to verify the mediating role of perceived green washing between product scarcity appeals of green products and consumers’ purchase intentions by using questions to initiate subjects’ perceived green washing.

## 4 Experiment 2

The purpose of experiment 2 was to verify whether the mediating effect of perceived green washing in product scarcity appeals on green product purchase intentions holds true.

### 4.1 Experimental design and subjects

A (scarcity group vs. control group) single factor experimental design was used for experiment 2. The same experimental method as in experiment 1 was used, and the stimuli and materials were changed, with “recyclable paper bottles” selected as the stimuli. In this experiment, 104 undergraduates from a university in southern China were used as subjects, and the respondents were divided into a scarcity group and a control group. Samples that did not meet the requirements were excluded, and 100 valid samples were finally retrieved (*M*_age_ = 24.88, *SD*_age_ = 3.72; 52% female). The results of the collected data showed that the scarcity group was 58 and the control group was 42.

### 4.2 Experimental procedures

The experiment began by randomly assigning subjects to the two groups mentioned above, divided into a scarcity group and a control group. Each subject participating in this experiment received a small gift after completing the experimental task independently. We used a role-playing method to have subjects read a self-compiled material in order to manipulate their perceived green washing toward buying green products with scarcity appeals.

This experiment was consistent with experiment 1’s manipulation of subjects’ perceptions of green product scarcity appeals and used a marketing scenario to initiate subjects’ perceived green washing. After reading the material, in order to determine the validity of the experimental material, subjects completed the questionnaire questions used in our adopted study by White and Peloza (2009) and Liu et al. (2017) and measured using a 5-point Likert scale. First, after reading the material, the subjects continued to rate their perceived emotions on a five-point scale (1 = not at all scarce, 5 = very scarce). The subjects also had to answer the questions in the questionnaire regarding the perceived green washing scale and the green product purchase intention scale, and choose the corresponding score for each statement according to the degree of agreement after reading the materials (1 = strongly disagree, 5 = strongly agree).

### 4.3 Results

Experiment 2 first verified whether the main effect of product scarcity appeals on perceived green washing was valid. The respondents of this study were first divided into a scarcity group and a control group, and the results of the collected data showed that there were 52 respondents in the scarcity group and 48 respondents in the control group. The hypotheses were tested by one-way ANOVA. In order to exclude the influence of control variables such as gender, age and education on the experimental results, we added gender, age and education together with product scarcity appeals as independent variables to the model. The results of descriptive statistics showed that respondents in the scarcity group (*M*_scarcity_ = 3.307, *SD*_scarcity_ = 0. 859) had a higher mean value of perceived green washing compared to respondents in the control group (*M*_control_ = 2.367, *SD*_control_ = 0.878).

The significance of the corrected model *p* = 0.000 < 0.05 showed that the current model had independent variables that had a significant effect on the dependent variable. The model fit was 0.125, which met the general linear model general criteria and the model fit was good. The results of the between-subjects effect test showed that in the model, there was no significant effect of gender on perceived green washing (*F* = 1.287, *p* = 0.26 > 0.05), no significant effect of age on perceived green washing (*F* = 0.351, *p* = 0.555 > 0.05), and no significant effect of education on perceived green washing (*F* = 1.463, *p* = 0.23 > 0.05), indicating that none of the control variables had a significant effect on perceived green washing. And product scarcity appeals had a significant effect on perceived green washing (*F* = 14.007, *p* = 0.000 < 0.05).

The second step was taken in order to verify whether the mediating effect of perceived green washing in the product scarcity appeals on the willingness to purchase green products holds true. We used gender, age, and education as control variables, product scarcity as independent variables, perceived green washing as mediating variables, and purchase intention as dependent variables, and analyzed the mediating effect of perceived green washing in product scarcity appeals on green product purchase intention by using the PROCESS program of SPSS macro program developed by Hayes, selecting model 4. And the bootstrap test results were also checked, the confidence interval was 95%, the sample size was chosen to be 5,000, and the sampling method was the non-parametric percentile method with selection bias correction. The output of PROCESS contained two parts: the regression coefficient test results and the bootstrap test results of the indirect effect, and the final mediating effect test results were summarized in Table 2.

**Table 2:** The mediating effect of perceived green washing.

Variables	Perceived green washing			Purchase intention		
	*B*	*se*	*t*	*B*	*se*	*t*
Constant	4.970	0.447	11.111^***^	2.607	0.584	4.467^***^
Perceived green washing				−0.405	0.088	−4.583^***^
Product scarcity appeals	0.934	0.175	5.322^***^	−0.409	0.172	−2.378^*^
Gender	−0.325	0.173	−1.872	0.064	0.152	0.420
Age	−0.024	0.078	−0.313	−0.131	0.067	−1.945
Education	−0.064	0.063	−1.011	−0.011	0.054	−0.199
*R*	0.511			0.610		
*R-sq*	0.261			0.372		
*F*	8.382^***^			11.121^***^		

^***^p < 0.001, ^**^p < 0.01, ^*^p < 0.05.

As can be seen from Table 2, when perceived green washing was the dependent variable, product scarcity appeals had a significant positive effect on perceived green washing (*B* = 0.934, *t* = 5.322, *p* < 0.001). When purchase intention was the dependent variable, perceived green washing had a significant negative effect on purchase intention (*B* = −0.405, *t* = −4.583, *p* < 0.001) and product scarcity appeals still had a significant negative effect on purchase intention (*B* = −0.409, *t* = −2.378, *p* < 0.05), indicating that perceived green washing had a significant partially mediated effect, product scarcity appeals can either have a negative effect on green product purchase intention directly or through the partially mediated effect of perceived green washing on green product purchase intention.

Further, based on PROCESS, which gave the model test for perceived green washing as an outcome variable, it can be concluded that the *R*-value of the model when perceived green washing was the outcome variable is 0.511, *p* < 0.001, which was statistically significant, and the regression coefficient *B* = 0.934, which had a significant effect (*p* < 0.001), with a 95% CI of (0.186, 0.607) and an interval not containing 0, indicating that product scarcity appeals had a positive correlation on perceived green washing.

Subsequent model testing with purchase intention as the outcome variable yields an *R*-value of 0.610, *p* < 0.001, indicating a statistically significant model when purchase intention was the outcome variable, with regression coefficient *B* (perceived green washing vs. purchase intention) = −0.405, with a significant effect (*p* < 0.001) and 95% CI of (−0.580, −0.229). Regression coefficient *B* (product scarcity appeals vs. purchase intention) = −0.409, significant (*p* < 0.05), 95% CI (−0.751, −0.068), interval did not contain 0. Therefore, it can be concluded that perceived green washing had a mediating effect, and perceived green washing was a partial mediating effect.

### 4.4 Discussion

Experiment 2 once again confirmed the relationship between product scarcity appeals and perceived green washing. By initiating subjects’ perception of product scarcity, it was tested that the perceived green washing in this scenario was consistent with hypothesis 1 of this paper, i.e., subjects had higher perceived green washing for green products with product scarcity appeals. Hypothesis 2 was tested again in experiment 2, where a question was asked to initiate subjects’ perceived green washing to verify the mediating role of perceived green washing between product scarcity appeals of green products and consumers’ purchase intentions.

## 5 Experiment 3

The purpose of experiment 3 was to verify that motivation of impression management moderated the mediating role of perceived green washing in product scarcity appeals on green product purchase intentions.

### 5.1 Experimental design and subjects

A (scarcity group vs. control group) single factor experimental design was used for experiment 3. The same experimental method as experiment 1 was used, but the stimuli and materials were changed, and “biodegradable plastic” was selected as the stimulus. In this experiment, 112 undergraduates from a university in southern China were used as subjects, and the respondents were divided into a scarcity group and a control group. The subjects who did not fill in the required samples were excluded, and 100 valid samples were finally obtained (*M*_age_ = 23.91, *SD*_age_ = 3.29; 51% female). The results of the collected data showed that the scarcity group was 61 and the control group was 39.

### 5.2 Experimental procedures

The subjects were first randomly assigned to the two groups mentioned above, divided into a scarcity group and a control group. Each subject participating in this experiment received a small gift after completing the experimental task independently. A role-playing approach was used to have the subjects read a self-compiled material to manipulate the effect of motivation of impression management on the purchase of a green product with a product scarcity appeal.

This experiment was consistent with experiment 1’s manipulation of subjects’ perceptions of green product scarcity appeals and used a marketing scenario to initiate subjects’ motivation of impression management. After reading the material, in order to determine the validity of the experimental material, subjects completed the questionnaire questions used in our adopted study by White and Peloza (2009) and Liu et al. (2017) and measured using a 5-point Likert scale. First, after the subjects read the material, they were given a five-point scale for the relevant emotion they felt (1 = not at all scarce, 5 = very scarce). The subjects were then asked to answer questions on the scales of motivation of impression management, perceived green washing, and purchase intentions about green products. The corresponding scores were selected according to the level of agreement after reading the material and the level of agreement with each statement (1 = strongly disagree, 5 = strongly agree).

### 5.3 Results

The results of descriptive statistics showed that respondents in the scarcity group (*M*_scarcity_ = 3.111, *SD*_scarcity_ = 1.062) had higher mean values of motivation of impression management compared to respondents in the control group (*M*_control_ = 2.712, *SD*_control_ = 1.054). Then we used gender, age, and education as control variables, product scarcity appeals as independent variables, motivation of impression management as moderating variables, perceived green washing as mediating variables, and green product purchase intention as dependent variables, and used Model 14 of the PROCESS program of the SPSS macro program developed by Hayes to analyze the effect of perceived green washing in the product scarcity appeals on green product purchase intention. The final results of the moderating effect test were shown in Table 3.

**Table 3:** The moderating effect of motivation of impression management.

Variables	Purchase intention				Perceived green washing			
	*β*	*SE*	*t*	*p*	*β*	*SE*	*t*	*p*
Constant	4.108	0.431	9.521	0.000	0.707	0.424	1.669	0.098
Product scarcity appeals	−0.524	0.191	−2.740	0.044	0.805	0.167	4.814	0.000
Motivation of impression management	0.102	0.079	1.290	0.200				
Gender	0.070	0.170	0.411	0.682	0.367	0.163	2.252	0.067
Age	−0.196	0.076	−2.591	0.061	−0.099	0.075	−1.328	0.188
Education	0.003	0.061	0.054	0.957	0.036	0.061	0.596	0.553
Perceived green washing	0.382	0.105	3.655	0.100				
Perceived green washing * Motivation of impression management	0.079	0.086	0.917	0.004				
Sample	100				100			
*R*	0.613				0.493			
*R* ^2^	0.376				0.243			
*F*	*F* = 7.908, *p* = 0.000				*F* = 7.605, *p* = 0.000			

The control variables gender (*B* = 0.070, *t* = 0.411, *p* > 0.05) had no significant effect, age (*B* = −0.196, *t* = −2.591, *p* > 0.05) had no significant effect, and education (*B* = 0.003, *t* = 0.054, *p* > 0.05) had no significant effect on willingness to purchase green products. Perceived green washing had no significant effect on green product purchase intention (*B* = 0.382, *t* = 3.655, *p* > 0.05). Motivation of impression management had no significant effect on green product purchase intention (*B* = 0.102, *t* = 1.290, *p* > 0.05). The effect value of the interaction term of perceived green washing and motivation of impression management on green product purchase intention is 0.079, and the corresponding significance *p* < 0.05 indicated that the interaction term of perceived green washing and motivation of impression management had a significant effect on green product purchase intention, i.e., the moderating effect of motivation of impression management on perceived green washing in product scarcity appeals on green product purchase intention was valid.

Further, when motivation of impression management was at a low level, the negative effect of perceived green washing on green product purchase intention was not significant with a 95% CI of (−0.481, 0.081) and an interval containing 0. When motivation of impression management was at a medium level, the effect of perceived green washing on green product purchase intention changed from negative to positive with *B* = 0.045 and a 95% CI of (0.122, 0.521), and the interval did not contain 0. When motivation of impression management was at a high level, the positive effect of perceived green washing on green product purchase intention was strongest with *B* = 1.255, 95% CI of (0.159, 0.682), and the interval did not contain 0. When motivation of impression management increases by one unit, the effect of product scarcity appeals on green product purchase intention through perceived green washing increased by 0.524 units. Thus, it can be seen that motivation of impression management moderated the effect of perceived green washing on the purchase intention of green products, and when motivation of impression management was at a high level, purchase intention was increased through the moderating effect.

### 5.4 Discussion

Experiment 3 tested that motivation of impression management in this scenario moderated the mediation of perceived green washing in product scarcity appeals on green product purchase intentions by initiating subjects’ motivation of impression management, which was consistent with hypothesis 3 of this paper. i.e., when subjects in the scarcity group had higher motivation of impression management compared to the control group, perceived green washing in product scarcity appeals would be lower, thus validating hypothesis 3. Experiment 3 also verified that motivation of impression management moderated the negative effect of product scarcity appeals on green product purchase intention by using questions to initiate subjects’ motivation of impression management.

## 6 General discussion

This study examines the fact that when green products express product scarcity appeals, consumers do not necessarily pay for them. In fact, green products are different from common products, and purchasing green products often reflects an individual’s pro-sociality, which belongs to designated morality and conforms to the category of morality, with the dual attributes of altruism and self-interest. In the past, studies have mainly explored how ordinary products can be marketed virtually through product scarcity appeals, or even converted into phantom alternatives, but few studies have explored how green products can be marketed virtually. Moreover, because green products have dual attributes, their virtual marketing may not have the same effect on them. On this basis, this study examines the effect of product scarcity appeals on the purchase intention of green products in a green product purchase context, and explores its intrinsic mechanisms and boundaries.

In addition, perception of deceptive increases consumers’ green consumption confusion and green perception risk, and affects consumers’ trust in green products. Therefore, the level of consumers’ perceived green washing affects their green purchase intention; the lower the perceived green washing, the higher the consumers’ purchase intention; the higher the perceived green washing, the lower the consumers’ purchase intention. Perceived green washing plays a mediating role in the effect of product scarcity appeals on purchase intention. In addition, motivation of impression management is a motivation to show its pro-social individual attributes to others. That is, consumers may perceive purchasing green products as a way to gain status associated with pro-social behavior and to show others that they are pro-social individuals. Consumers’ own motivation of impression management may lead them to engage in pro-social altruistic behaviors, such as showing more interdependent self-image in group purchasing scenarios. Therefore, this thesis verifies through research that when consumers’ motivation of impression management is high, they will reduce their perceived green washing of green products with product scarcity appeals as a strategy, thus increasing their willingness to purchase such products.

Regarding the results of the experiment, experiment 1 preliminarily verifies that product scarcity appeals have a negative effect on green product purchase intentions. Experiment 2 again verified the inhibitory effect of product scarcity appeals on purchase intentions, and also verified the mediating role of perceived green washing, specifically, products with high scarcity appeals can effectively enhance consumers’ perceived green washing compared to products with low scarcity appeals, which in turn reduces consumers’ green product purchase intentions. Experiment 3 found that the moderating effect of motivation of impression management on perceived green washing in product scarcity appeals on green product purchase intentions, when products with scarcity appeals have high consumer motivation of impression management, consumers’ perceived green washing on product scarcity appeals will be reduced and they will have positive purchase intentions for green products.

### 6.1 Theoretical implications

First, this study extends the research on product scarcity appeals of green products. Product scarcity appeals are often found in virtual products, where consumers perceive greater risk due to the vague product concept and lack of physical presence, and are thus more likely to produce perceived green washing in certain contexts (Li D.J. et al., 2015; Li D.Y. et al., 2015; Liu et al., 2017). Product scarcity appeals are an important concept in virtual marketing, and previous studies have focused on the impact of product scarcity appeals on ordinary product purchase intentions, and less on the effect of product scarcity appeals in green product purchase contexts; in fact, green products are different from ordinary products, and the purchase of green products often reflects the pro-sociality of individuals, which is in line with the moral category and has the dual attributes of altruism and self-interest. Past research has focused on how ordinary products can be marketed in a false sense through product scarcity appeals, or even transformed into phantom alternatives, i.e., “products that are not available at the time of purchase” (Young et al., 2010). However, few studies have explored how phantom marketing is conducted for green products, which are different from ordinary products in that they have dual attributes, i.e., pro-social moral attributes and self-interest utility attributes, and their phantom marketing may have different effects on them. This study explores the impact of product scarcity appeals on the purchase intention of green products in the context of green product purchase, and explores the intrinsic mechanisms and boundaries.

Second, this study extends the findings of previous studies on perceived green washing on green product purchase intentions. It has been confirmed that in Chen and Chang (2013) found that deceptiveness increases consumer confusion about green consumption and perceived risk of greenness, affecting consumer trust in green products. Therefore, the level of consumers’ perceived green washing affects their green purchase intentions; the lower the perceived green washing, the higher the consumers’ purchase intentions; the higher the perceived green washing, the lower the consumers’ purchase intentions. Therefore, if green products are promoted using the product scarcity appeal, consumers may easily perceive this promotion as a strategy for companies to promote themselves as green to gain social reputation, rather than really wanting to produce green products, because these products are “scarce” or even high-priced, and consumers may think that companies are not going to produce large quantities of these products for environmental improvement, and thus consumers may have a perceived green washing, which affects their willingness to buy the products.

Third, this study has several implications in terms of exploring the mechanism of the role of consumer motivation of impression management on the purchase intention of green products. Motivation of impression management is a motivation to demonstrate to others that they are pro-social individuals. That is, consumers perceive purchasing green products as a way to gain status associated with prosocial behavior and to demonstrate to others that they are prosocial individuals. In fact, motivation of impression management is present in every individual, but the strength of motivation of impression management is not consistent for different individuals. It has been suggested that when people are able to leave a positive self-image to others when they engage in pro-social behaviors, then this impression management motivation motivates consumers to engage in more pro-social behaviors (Wu, 2014). The interdependent self, which views oneself as a member of a social relationship and emphasizes the importance of an individual’s relationship with others and the interdependence of both, is a completely different self-concept from the independent self (Abele et al., 2021). In studies on social interaction aspects, motivation of impression management motivates people to try to change the impression they form in the minds of others; consumers’ own motivation of impression management motivates them to engage in pro-social altruistic behaviors, for example, in group buying scenarios, consumers will show more interdependent self-image (Bolino et al., 2008). Therefore, this thesis verifies through research that when consumers’ motivation of impression management is high, they will reduce their perceived green washing toward green products that use product scarcity appeals as a strategy, thus increasing their willingness to purchase such products.

### 6.2 Managerial implications

First, this study provides a theoretical basis for companies to avoid the negative effects of advertising when promoting green products. Advertising is the most direct and effective promotional tool used by companies to promote green products or services, and it can significantly influence consumers’ attitudes and behaviors toward such products. In addition to making advertising promotional, attention needs to be paid to avoiding negative effects of advertising, which is not only an evergreen topic in advertising and consumer behavior, but also a problem that needs to be solved by companies in general. This study finds that product scarcity appeals can make consumers easily perceive deceptive advertising messages and reduce their willingness to buy, which also applies to green product consumption. Therefore, companies should avoid monotonously and emphatically expressing product scarcity appeals in advertisements promoting green products, and instead promote the unique environmental attributes of green products to enhance consumers’ willingness to purchase green products.

Second, companies should highlight “green” rather than “green washing” when using the green product logo. The study found that for consumers who are highly concerned about environmental issues, the more familiar they are with the brand, the higher their enthusiasm for consumption if the product has a green certification logo; however, when the product packaging does not have an eco-label, brand familiarity has no significant impact on purchase intentions, but consumers will have perceived green washing, thus reducing the desire to buy the product. For consumers who are less concerned about environmental issues, when products with green logos and other content that can reflect their environmental characteristics, consumers will change their inner discriminatory results about their quality and green washing behavior to the point of weakening their own impulse to consume. Therefore, when companies try to use logos to prove that their products are “green,” they should also convey information about the quality of their products to consumers.

Third, this study finds that motivation of impression management can play an important moderating role when firms promote green products using scarcity appeals. Specifically, for consumers with high motivation of impression management, consuming green products will make them feel that their moral image is improved, and thus they tend to prefer green products that are promoted using scarcity appeals. Therefore, when using product scarcity appeals, companies should design different advertising contents according to the consumer portrait and different advertising audiences, so that consumers with high motivation of impression management can experience the product scarcity appeals expressed in the advertising, thus achieving the marketing effect that companies want.

### 6.3 Limitations and future research

In terms of study limitations. Firstly, the experimental subjects who participated in this study were mainly undergraduate and graduate students in a university. Therefore, the subjects were relatively homogeneous in terms of education, age and education level, and the purchase of green products occurred in different groups. Therefore, in future studies, the scope of subjects can be further extended to different consumer groups to improve the generalizability and practicability of the findings of this study. Secondly, this study investigates how product scarcity appeals of green products affect consumers’ purchase intentions in the context of green marketing communication, and what are the potential mechanisms and boundary conditions involved. There are many types of product scarcity appeals, such as supply and demand product scarcity appeals, limited and time-limited product scarcity appeals, environmental and human product scarcity appeals, explicit and implicit product scarcity appeals, and so on. Therefore, the research products in this paper are relatively limited. Lastly, the moderating effect in this paper also only detects motivation of impression management, while in fact many factors such as product, information, personality, and context may have moderating effects, and this paper does not test for multivariate moderating effects. The use of experimental quantitative research methods in this paper is the best way to manipulate the variables and test the causal relationship between the independent and dependent variables. Although this paper uses different experimental goods and contexts for the study, the effects of product scarcity appeals are very broad compared to the proposed model in the paper. For example, the different scarcity effects caused by the situation where the supply of the good does not change on the supply side and the situation where the product cannot meet the market demand can have different effects on consumers’ value judgments and purchase intentions. Therefore, the impact of two different scarcity causes and scarcity types on consumers’ willingness to purchase green products can be further investigated in depth.

In terms of future research directions. Although this paper combines qualitative and quantitative mixed methods, due to the inadequacy of the research method, different methods are needed to further enhance the reliability and validity of the study in future studies. First is to strengthen qualitative research. In reviewing previous research literature, it was found that there is a serious lack of qualitative research on product scarcity appeals. This therefore greatly limits the impact of explored product scarcity appeals, which can also affect the discovery of new perspectives and mechanisms, as well as limit the expansion of theoretical constructs and marketing practices. Therefore, future research is needed to reinforce the existing research on the impact of product scarcity appeals using, for example, rooting theory, focus interviews, and phenomenological observation. Different qualitative research methods should be utilized to explore the potential influencing mechanisms in a deeper and more comprehensive way. The second is the full use of advanced methods and techniques. With the development, advanced methods and techniques are widely used in many studies, such as implicit attitude measurement, neural network techniques, etc. It has been confirmed from practice that the research findings obtained by using such research methods are scientifically rich. Therefore, in further research in the future, advanced methods and techniques, such as eye-tracking and big data mining, should be fully used to obtain more credible and valid research findings. Third, improve the traditional research methods. The review reveals that the current research on product scarcity appeals is still mainly based on traditional experimental research methods. The traditional methods can be further enhanced and improved in future studies, such as expanding the product range, simulating consumption situations, enhancing the experimental sample size and cross-cultural studies to improve the existing data collection methods. It is believed that changes in traditional experimental methods can improve the external validity of research models and findings.

## Data availability statement

The raw data supporting the conclusions of this article will be made available by the authors, without undue reservation.

## Ethics statement

The studies involving human participants were reviewed and approved by the Ethics Committee of the School of Management, Jinan University, China. The patients/participants provided their written informed consent to participate in this study. Written informed consent was obtained from the individual(s) for the publication of any potentially identifiable images or data included in this article.

## Author contributions

SY: Conceptualization, Data curation, Formal analysis, Funding acquisition, Methodology, Project administration, Resources, Supervision, Writing − original draft, Writing − review & editing. GL: Conceptualization, Data curation, Formal analysis, Investigation, Methodology, Project administration, Writing − original draft, Writing − review & editing, Validation, Supervision. YL: Formal analysis, Investigation, Validation, Visualization, Writing − original draft, Writing − review & editing. ZL: Formal analysis, Investigation, Methodology, Writing − original draft, Writing − review & editing. YS: Conceptualization, Formal analysis, Methodology, Writing − original draft, Writing − review & editing. ZH: Conceptualization, Methodology, Project administration, Supervision, Writing − review & editing, Validation.
